# Association of Resolved Low-Lying Placentation With Risk of Postpartum Hemorrhage

**DOI:** 10.1097/og9.0000000000000042

**Published:** 2024-11-07

**Authors:** Sara Ornaghi, Elisabetta Colciago, Laura Montelisciani, Francesca Arienti, Federica Fernicola, Alessandra Abbamondi, Sofia Giani, Simona Fumagalli, Laura Antolini, Isadora Vaglio Tessitore, Giulia Zangheri, Elena Gatti, Michele Vignali, Clelia Callegari, Andrea Sala, Cristina Plevani, Maddalena Smid, Mirko Pozzoni, Maria Castoldi, Sara Benedetti, Mario G. Meroni, Camilla Bulfoni, Anna Catalano, Sara Consonni, Anna Fichera, Elisa Fabbri, Patrizia Vergani, Anna Locatelli

**Affiliations:** Unit of Obstetrics, Foundation IRCCS San Gerardo dei Tintori, the University of Milan-Bicocca, School of Medicine and Surgery, and the 4 Center of Bioinformatics, Biostatistics, and Bioimaging, School of Medicine and Surgery, University of Milan-Bicocca, Monza, the Unit of Obstetrics and Gynecology, Vittorio Emanuele III Hospital, ASST Brianza, Carate Brianza, the Unit of Obstetrics and Gynecology, Macedonio Melloni Hospital, ASST Fatebenefratelli Sacco, the Unit of Obstetrics and Gynecology, IRCCS San Raffaele Scientific Institute, Vita-Salute San Raffaele University, the Unit of Obstetrics and Gynecology, ASST Grande Ospedale Metropolitano Niguarda, and the Unit of Obstetrics and Gynecology, Vittore Buzzi Hospital, ASST Fatebenefratelli Sacco, University of Milan, School of Medicine and Surgery, Milan, the Unit of Obstetrics and Gynecology, Alessandro Manzoni Hospital, ASST Lecco, Lecco, the Unit of Obstetrics and Gynecology, Poliambulanza Foundation Hospitals, and the Unit of Obstetrics and Gynecology, Spedali Civili Hospital, Department of Clinical and Experimental Sciences, University of Brescia, Brescia, Italy.

## Abstract

**OBJECTIVE:**

To evaluate whether individuals who have a placenta previa or low-lying placenta that resolves before delivery are at increased risk of postpartum hemorrhage and postpartum hemorrhage–related morbidity.

**METHODS:**

This was a prospective, multicenter, 1:3 matched cohort study of pregnant individuals with singleton gestations diagnosed with low placentation by transvaginal scan at 19 to 23 weeks of gestation between January 2021 and December 2023 at nine academic maternity centers (exposed participants). Unexposed participants were those with a normally located placenta, matched in a 1:3 ratio according to parity. Resolution of low placentation was diagnosed when placenta was at 20 mm or more from the internal os. Individuals with fetal anomalies, hematologic disorders, therapeutic anticoagulation, placenta accreta spectrum disorder, vasa previa, persistent low placentation at birth, and delivery at a nonenrolling center were excluded. Primary outcome was postpartum hemorrhage of 1,000 mL or more. Secondary outcomes included postpartum hemorrhage of 1,500 mL or more, use of second-line uterotonic drugs, blood transfusions, additional procedures to control bleeding, intensive care unit admission, and hospital stay for more than 7 days. Multivariable logistic regression adjusted for confounders was used to estimate independent associations with outcomes.

**RESULTS:**

The study population included 182 exposed and 589 unexposed participants. Individuals with resolved low placentation had higher rates of smoking (*P*=.024), prior dilation and curettage (*P*=.012), posterior placenta (*P*<.001), and induction of labor (*P*=.038). Multivariate logistic regression analysis adjusted for confounders showed that exposed people had higher odds of postpartum hemorrhage of 1,000 mL or more (13.2% vs 4.1%, adjusted odds ratio [aOR] 3.1) compared with unexposed people and of use of second-line uterotonic drugs (28.0% vs 12.4%, aOR 2.69) and tranexamic acid (16.5% vs 7.5%, aOR 2.19), as well as hospital stay longer than 7 days (11.5% vs 3.4%, aOR 2.63).

**CONCLUSION:**

Individuals with resolved low placentation are at increased risk of postpartum hemorrhage and related complications compared with those who always had a normally located placenta.

## Abstract 2

Pregnant individuals with resolved low placentation are at increased risk of postpartum hemorrhage and related complications compared with those who always had a normally located placenta.

Placenta previa and low-lying placenta are conditions characterized by placental implantation in the lower uterine segment and can be referred to collectively as low placentation. In placenta previa, the leading placental edge overlaps the internal os, whereas it is located 1–20 mm from the internal os in low-lying placenta.^1,2^ Assessment of the placental edge location in relation to the internal os is ideally performed by transvaginal ultrasonography, which is the gold standard technique for accurately assessing low placentation by measuring the distance between the placental edge and the internal os (internal os distance).^3^ Low placentation is diagnosed in 5–11% of pregnancies at the time of the midtrimester scan. However, the prevalence of this condition decreases to 0.3–1% at delivery.^4,5^ This is attributable to resolution of low placentation as gestation progresses, likely subsequent to trophotropism, with atrophy of the placental margin at the lower uterine segment, growth of cranial placental regions toward the more vascularized uterine fundus, and differential development of the lower uterine segment.^6,7^

Persistent low placentation at the time of delivery is known to increase the risk of postpartum hemorrhage.^8–15^ However, the risk of postpartum hemorrhage when low placentation undergoes resolution before delivery is less certain.^16–23^ Even if resolution occurs, it is plausible that initial placental implantation in the lower uterine segment may increase its vascularity, possibly increasing the risk for postpartum hemorrhage as a result of inadequate contraction of these vascular beds at delivery. Available data are methodologically limited because of the use of transabdominal instead of transvaginal ultrasonography,^17,20,23^ varying internal os distance cutoffs (eg, 10, 25, and 30 mm)^17,19–21^ for diagnosing low placentation, inadequate assessment of postpartum hemorrhage,^16–19,21–23^ and retrospective design.^16,17,19,21–23^ For these reasons, resolved low placentation is not currently included as a risk factor for postpartum hemorrhage in most guidelines issued on the topic.^8–10,12^ Only the guideline recently issued by the Society of Obstetricians and Gynecologists of Canada includes resolved low-lying placenta as a medium risk factor in its postpartum hemorrhage admission risk assessment tool.^11^

Because most people with initial low placentation will have a resolved placenta by term^4,5,24,25^ and postpartum hemorrhage still represents a leading direct cause of severe maternal morbidity and mortality in Italy, as well as worldwide,^26,27^ high-quality evidence on the risk of postpartum hemorrhage in individuals with resolved low placentation is needed to inform appropriate postpartum hemorrhage risk stratification. We sought to perform a prospective cohort study to assess the risk of postpartum hemorrhage and related morbidity in individuals with resolved low placentation compared with those with normally located placenta throughout gestation.

## METHODS

This is a prospective, multicenter cohort study conducted at nine academic maternity centers located in Northern Italy. The research sites are members of a regional maternal–fetal medicine network and share similar institutional protocols for managing postpartum hemorrhage,^10^ as well as similar rates of postpartum hemorrhage (4%) and of cesarean delivery (19–20%). All participating centers perform transvaginal ultrasonography on the basis of risk factors for preterm birth or because of suspected low placentation or shortened cervix by transabdominal scan, and none perform transvaginal ultrasonography universally at the midtrimester scan. All transvaginal ultrasonograms are performed by expert maternal–fetal medicine physicians after voiding, using a straight-line approach with no fundal pressure to define the extent of the placental edge beyond the internal os in the case of placenta previa or the internal os distance in the case of a low-lying placenta. Three measurements were taken to ensure accuracy, with the shortest one being recorded.^2^

Between January 2021 and December 2023, we consecutively enrolled all pregnant individuals with singleton gestations diagnosed with low placentation by transvaginal ultrasonography between 19 0/7 and 23 6/7 weeks of gestation. These individuals (exposed group) were matched in a 1:3 ratio to individuals with a normally located placenta diagnosed at the same gestational age range, with matching based on parity (unexposed group). Normal location of the placenta was confirmed by transvaginal ultrasonography. *Resolution of low placentation* was defined as occurring when the leading edge of the placenta was at more than 20 mm from the internal os as measured by transvaginal ultrasonography at a subsequent scan. Timing of follow-up transvaginal ultrasonography was planned according to the maternal–fetal medicine physician treating the woman, with a first follow-up scan usually at 26–30 weeks of gestation and subsequent scans every 2–3 weeks until delivery in case of persistent low placentation.^28^ Data on sociodemographic characteristics, including race because of its relation with postpartum hemorrhage and related morbidity, medical and obstetric history, pregnancy course, ultrasonography data, labor and birth outcomes, and neonatal outcomes, were collected with anonymous electronic case report forms. Individuals with multiple gestations, major fetal anomalies, hematologic disorders predisposing to bleeding (eg, von Willebrand disease, hemophilia), therapeutic anticoagulation, antenatal suspicion of placenta accreta spectrum disorder, diagnosis of vasa previa, persistent low placentation at childbirth, and delivery at a nonenrolling center were excluded from the analyses.

The primary outcome was *primary postpartum hemorrhage*, defined as quantitative cumulative blood loss at delivery of 1,000 mL or more or accompanied by signs and symptoms of hypovolemia, including hypotension and tachycardia requiring intravenous fluid or blood product resuscitation, within 24 hours after birth.^29^ Blood loss at delivery was quantified with the use of graduated collection bags and visual aids^30^ for vaginal deliveries and with suction bottles and weighted surgical pads for cesarean deliveries. Secondary outcomes included 1) severe postpartum hemorrhage of 1,500 mL or more, 2) second-line uterotonic drugs (sulprostone, carboprost tromethamine, methylergonovine, and misoprostol) and tranexamic acid, 3) blood transfusions, 4) additional procedures to control bleeding (intrauterine balloon tamponade, dilation and curettage [D&C], compressive uterine or vascular sutures, uterine artery embolization, and hysterectomy), 5) maternal intensive care unit admission, and 6) hospital stay longer than 7 days.

Power analysis was conducted by means of the Pearson χ^2^ test comparing two independent proportions. Considering a baseline rate of primary postpartum hemorrhage among enrolling centers of 4%, we estimated a sample size of 159 exposed individuals and 477 unexposed individuals to detect a threefold increased risk of primary postpartum hemorrhage among individuals with resolved low placentation, with a power equal to 0.80 and a two-sided value of α=.05. Considering a potential attrition rate of 10%, the final sample size was 177 individuals in the case group and 531 individuals in the control group.

Continuous variables were described with mean and SD or median and interquartile range according to their distribution as defined by visual inspection, and qualitative variables were reported as count and frequency. Demographic, pregnancy, delivery, and neonatal characteristics were compared between groups with the Student *t* test or Mann–Whitney *U* test for continuous measures and χ^2^ or Fisher exact tests for categorical measures as appropriate. A multivariable logistic regression model was used to estimate associations with the outcomes of interest, adjusted for potential confounding variables, with unexposed participants as referent. Results were reported as adjusted odds ratio and 95% CI. A planned secondary analysis was performed that was based on timing of low placentation resolution: early resolution (before 33 weeks of gestation) and late resolution (at or after 33 weeks of gestation).

The study was approved by the IRB of the Brianza Ethic Committee (No. 3157, December 16, 2019), and written informed consent was obtained from all participants. Statistical analyses were performed using the open-source R 4.4. and STATA 16.

## RESULTS

**Fig. 1.**
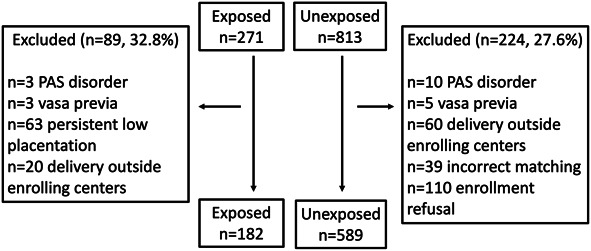
Flowchart of the study. The figure displays the number of women with low placentation (exposed) and normally located placenta (unexposed) at enrollment. Exclusion criteria are reported with the number of exposed and unexposed women excluded from the analyses. PAS, placenta accreta spectrum.

We enrolled 271 exposed and 813 unexposed participants (Fig. 1). Criteria assessment led to exclusion of 89 exposed individuals (32.8%) and 224 unexposed individuals (27.6%), resulting in 182 participants with resolved low placentation and 589 with a normally located placenta included in the analysis. Baseline characteristics and outcomes in retained and excluded exposed and unexposed individuals were similar.

*Ornaghi. Hemorrhage in Resolved Low Placentation. O&G Open 2024*.

Among the 182 exposed participants included in the analysis, 60 (33%) were diagnosed with placenta previa at the time of enrollment, with a median extension of the placenta beyond the internal os of 1 mm (minimum–maximum 0–34 mm). The remaining 122 participants (67%) had a low-lying placenta, with a median internal os distance of 12 mm (minimum–maximum 2–19 mm). Median gestational age at resolution was 31 weeks (interquartile range 29–35 weeks), with 68.7% of cases (n=125) of low placentation resolving before 33 weeks. Early resolution more frequently occurred among participants with initial low-lying placenta than those with placenta previa (n=96 [78.7%] vs n=29 [48.3%], *P*<.001).

Table 1 shows maternal demographic characteristics, obstetric history, and pregnancy course. Enrolled individuals were mostly non-Hispanic White (n=663, 86%) with no pregestational diseases (n=747, 96.9%). Patients with resolved low placentation displayed higher rates of smoking (7.7% vs 3.4%, *P*=.024), prior D&C (15.9% vs 9.0%, *P*=.012), and a placenta positioned on the posterior uterine wall (67.6% vs 46.0%, *P*<.001). In addition, these people were the only ones experiencing antenatal bleeding episodes requiring hospital admission (n=11 [6.0%], *P*<.001). Mean±SD gestational age at the first bleeding episode was 28.1±3.5 weeks of gestation, with six people (54.5%) having the first episode before 29 weeks of gestation.

**Table 1.** General Characteristics, Obstetric History, and Pregnancy Course

	Overall (N=771)	Unexposed (n=589)	Exposed (n=182)	*P*
Maternal age (y)	33.64±4.81	33.54±4.78	33.96±4.90	.298
Older than 35 y	283 (36.7)	210 (35.7)	73 (40.1)	.316
Non-Hispanic White	663 (86.0)	511 (86.8)	152 (83.5)	.328
Pregestational BMI (kg/m^2^)	23.25±4.44	23.23±4.66	23.30±3.65	.853
30 or higher	60 (7.8)	50 (8.5)	10 (5.5)	.246
Chronic pregestational disease*	24 (3.1)	19 (3.2)	5 (2.7)	.936
Smoking in pregnancy	34 (4.4)	20 (3.4)	14 (7.7)	.024
1st pregnancy	292 (37.9)	226 (38.4)	66 (36.3)	.671
Nulliparity	432 (56.0)	328 (55.7)	104 (57.1)	.795
Parity 3 or more	26 (3.4)	21 (3.6)	5 (2.7)	.765
Prior miscarriage	190 (24.6)	136 (23.1)	54 (29.7)	.089
Prior term pregnancy	350 (45.4)	267 (45.3)	83 (45.6)	1.000
Prior cesarean delivery	68 (8.8)	54 (9.2)	14 (7.7)	.643
Prior D&C	82 (10.6)	53 (9.0)	29 (15.9)	.012
Prior placental manual removal	8 (1.0)	5 (0.8)	3 (1.6)	.792
Prior myomectomy	8 (5.1)	4 (3.5)	4 (9.3)	.281
Current myomas	17 (2.2)	12 (2.0)	5 (2.7)	.778
ART-induced fertilization	45 (5.8)	33 (5.6)	12 (6.6)	.751
Pregnancy complications†	255 (33.1)	191 (32.4)	64 (35.2)	.551
Posterior placenta	394 (51.1)	271 (46.0)	123 (67.6)	<.001
Placental morphology anomalies‡	9 (1.2)	4 (0.7)	5 (2.7)	.061
RDS prophylaxis	6 (42.9)	0	6 (54.5)	.301

BMI, body mass index; D&C, dilation and curettage; ART, artificial reproductive techniques; RDS, respiratory distress syndrome.

Data are mean±SD or n (%) unless otherwise specified.

* Includes chronic hypertension and pregestational diabetes mellitus.

† Includes hypertensive disorders of pregnancy, gestational diabetes, cholestasis, thyroid disease, and fetal growth restriction.

‡ Includes bilobate placenta and succenturiate placenta.

Labor and delivery outcomes are shown in Table 2. Overall, 30.8% of participants (n=219) underwent induction of labor, with a higher rate among exposed than unexposed participants (37.4% vs 28.7%, *P*=.038), primarily for rupture of membranes at term more than 24 hours (39.1% vs 20.0%, *P*=.030). No differences were identified regarding rate of anemia at hospital admission for delivery, gestational age at birth, and mode of delivery (Table 2). Primary postpartum hemorrhage occurred in 48 individuals (6.2%), with a 13.2% rate among exposed people compared with 4.1% among unexposed people (*P*<.001). In addition, individuals with resolved low placentation had higher rates of severe postpartum hemorrhage (n=11 [6.0%] vs n=6 [1.0%], *P*<.001) and more frequently required second-line uterotonic drugs (n=51 [28.0%] vs n=73 [12.4%], *P*<.001), tranexamic acid (n=30 [16.5%] vs n=44 [7.5%], *P*=.001), and fibrinogen concentrate (n=4 [2.2%] vs n=1 [0.2%], *P*=.014). In addition, these participants displayed increased need of blood products (n=7 [3.8%] vs n=1 [0.2%], *P*<.001) and additional procedures to control bleeding (n=14 [7.7%] vs n=10 [1.7%], *P*<.001). Specifically, among the 14 people who required additional procedures, nine received intrauterine tamponade and eight received D&C, with four undergoing both procedures; among unexposed people, two had both uterine tamponade and D&C, and the remaining eight had only D&C. None of the enrolled individuals required uterine or vascular sutures, and no uterine artery embolizations were performed. One case of peripartum hysterectomy occurred in a 39-year-old person in her fourth pregnancy with a resolved low-lying placenta at 32 weeks of gestation. She underwent an emergency cesarean delivery for bleeding at 38 1/7 weeks of gestation, with a blood loss of 1,600 mL. On postpartum day 1, she experienced delayed postpartum hemorrhage with an additional blood loss of 1,300 mL; conservative measures were unsuccessful, and hysterectomy was performed. No participants were admitted to the intensive care unit. Overall, 41 people (5.3%) required a prolonged hospital stay longer than 7 days; this occurred more frequently among exposed than unexposed people (n=21 [11.5%] vs n=20 [3.4%], *P*<.001).

**Table 2.** Delivery and Postpartum Outcomes

Outcome	Overall (N=771)	Unexposed (n=589)	Exposed (n=182)	*P*
Hb less than 10.5 g/dL at admission for childbirth	45 (5.8)	37 (6.3)	8 (4.4)	.443
Admitted to labor	712 (92.3)	541 (91.9)	171 (94.0)	.439
Induction of labor*	219 (30.8)	155 (28.7)	64 (37.4)	.038
PROM more than 24 h	−52 (23.7)	−31 (20.0)	−25 (39.1)	.030
Maternal disease	−29 (13.2)	−20 (12.9)	−5 (7.8)	.991
Fetal growth or AF anomalies	−43 (19.6)	−36 (23.2)	−7 (10.9)	.068
Postterm	−25 (11.4)	−20 (12.9)	−5 (7.8)	.991
Labor	666 (86.6)	506 (85.9)	160 (88.9)	.367
Epidural analgesia	190 (24.6)	136 (23.1)	54 (29.7)	.116
Duration of 2nd stage (min)	73.14±75.63	71.70±76.27	77.66±73.67	.399
Gestational age at birth (wk)	39.14 (38.43, 40.00)	39.14 (38.29, 40.00)	39.14 (39.00, 40.00)	.818
Preterm birth before 37 wk	37 (4.8)	28 (4.8)	9 (4.9)	1.000
Cesarean delivery	144 (18.7)	113 (19.2)	31 (17.0)	.588
For bleeding	−3 (2.1)	−2 (1.8)	−1 (3.1)	
For fetal malpresentation	−18 (12.8)	−13 (11.8)	−5 (16.1)	
Perineal lacerations†	21 (3.3)	13 (2.7)	8 (5.3)	.205
3rd-stage care†	617 (98.4)	466 (97.9)	151 (100.0)	.155
Manual removal of the placenta†	21 (3.3)	15 (3.2)	6 (4.0)	.818
D&C†	18 (2.9)	10 (2.1)	8 (5.3)	.077
Blood loss (mL)	300.0 (200.0, 500.0)	300.0 (200.0, 500.0)	400.0 (300.0, 687.5)	<.001
Primary outcome				
Primary PPH 1,000 mL or more‡	48 (6.2)	24 (4.1)	24 (13.2)	<.001
Secondary outcomes				
Severe primary PPH 1,500 mL or more	17 (2.2)	6 (1.0)	11 (6.0)	<.001
2nd-line uterotonic drugs§	124 (16.1)	73 (12.4)	51 (28.0)	<.001
Tranexamic acid	74 (9.6)	44 (7.5)	30 (16.5)	.001
Fibrinogen	5 (0.6)	1 (0.2)	4 (2.2)	.014
Blood products	8 (1.0)	1 (0.2)	7 (3.8)	<.001
Need for additional procedures||	24 (3.1)	10 (1.7)	14 (7.7)	<.001
Hospital LOS 7 d or longer	41 (5.3)	20 (3.4)	21 (11.5)	<.001

Hb, hemoglobin; PROM, premature rupture of membranes; AF, amniotic fluid; D&C, dilation and curettage; PPH, postpartum hemorrhage; LOS, length of stay.

Data are n (%), mean±SD, or median (interquartile range) unless otherwise specified.

* Only for women admitted to labor; each woman could display more than one indication for induction of labor.

† Only for participants with vaginal delivery (n=627 total; n=151 resolved low placentation and n=476 normally located placenta). Third-stage care includes delayed cord clamping (in presence of neonatal and maternal well-being), 10 international units of oxytocin intramuscularly at the birth of the anterior shoulder, and controlled cord traction.

‡ Included individuals with primary PPH, defined as cumulative quantitative blood loss at delivery of 1,000 mL or more or accompanied by signs and symptoms of hypovolemia within 24 hours after birth.^29^

§ Includes sulprostone, carboprost tromethamine, methylergometrine, and misoprostol.

|| Includes intrauterine balloon tamponade, peripartum and postpartum surgery (D&C, vascular sutures, uterine compressive sutures, hysterectomy), and uterine artery embolization.

Findings of the univariate analysis for primary and secondary outcomes were confirmed with the multivariable logistic regression model adjusted for confounders, including smoking in pregnancy, prior D&C, admission for bleeding, and induction of labor (Table 3), with those with resolved low placentation experiencing a 3.1-fold higher odds of experiencing primary postpartum hemorrhage of 1,000 mL or more compared with unexposed people.

**Table 3.** Multivariate Regression Analysis for Primary and Secondary Outcomes in Individuals With Resolved Low Placentation Compared With Those With Normally Located Placenta Throughout Gestation

	cOR (95% CI)	aOR (95% CI)*	
Primary outcome		
Primary PPH 1,000 mL or more†	3.57 (1.96–6.50)	3.11 (1.60–6.01)	
Secondary outcomes			
Severe primary PPH 1,500 mL or more‡	6.16 (2.28–18.45)	—	
2nd-line uterotonic drugs	2.75 (1.83–4.12)	2.69 (1.72–4.18)	
Tranexamic acid	2.45 (1.47–4.01)	2.19 (1.24–3.80)	
Blood products‡	20.91 (3.59–534.59)	—	
Need of additional procedures‡	4.79 (2.09–11.41)	—	
Hospital LOS 7 d or longer	3.70 (1.95–7.07)	2.63(1.30–5.27)	

cOR, crude odds ratio; aOR, adjusted odds ratio; PPH, postpartum hemorrhage; LOS, length of stay.

* Adjusted for smoking in pregnancy, history of dilation and curettage, antepartum admission for bleeding, and induction of labor.

† Included people with *primary PPH*, defined as cumulative quantitative blood loss at delivery of 1,000 mL or more or accompanied by signs and symptoms of hypovolemia within 24 hours after birth.^29^

‡ To avoid overfitting, estimation of aORs has been omitted for these outcomes because their counts are too few to be adjusted for the confounders included in the regression analysis.

No differences were identified between the two groups regarding neonatal outcomes (Table 4). However, counts of 5-minute Apgar scores less than 7 and neonatal intensive care unit admission were too few, and the study was underpowered to compare these findings. There was one death on postnatal day 2 of a neonate born at 37 weeks of gestation to a mother with resolved low placentation at 29 weeks of gestation and preeclampsia without severe features, who underwent an emergency cesarean delivery for placental abruptio and fetal bradycardia.

**Table 4.** Neonatal Outcomes

Outcome	Overall (N=771)	Unexposed (n=589)	Exposed (n=182)	*P*
Birth weight (g)	3,280.3±448.6	3,275.3±458.0	3,296.6±417.6	.576
More than 4,000	32 (4.2)	25 (4.2)	7 (3.8)	.982
Male neonate	418 (54.2)	322 (54.7)	96 (52.7)	.712
5-min Apgar score less than 7*	2 (0.3)	1 (0.2)	1 (0.5)	—
NICU admission*	21 (2.7)	19 (3.2)	2 (1.1)	—

NICU, neonatal intensive care unit.

Data are mean±SD or n (%) unless otherwise specified.

* *P* not presented because counts for these events are too few.

People with early resolution of low placentation showed rates of postpartum hemorrhage similar to those with late resolution (postpartum hemorrhage 1,000 mL or more, 11.2% vs 17.5%, *P*=.349; postpartum hemorrhage 1,500 mL or more, 6.4% vs 5.3%, *P*=1.000).

Multivariate logistic regression analysis adjusted for confounders identified a 2.5- and 4.6-fold heightened odds of primary postpartum hemorrhage in participants with early and late resolution compared with unexposed participants, respectively (Appendix 1, available online at http://links.lww.com/AOG/D893).

## DISCUSSION

We found that pregnant individuals with resolved low placentation are at increased risk of postpartum hemorrhage compared with those with a normally located placenta throughout pregnancy. Resolved low placentation was also associated with higher odds of needing additional uterotonic and antifibrinolytic medication and of requiring a prolonged hospital stay.

These findings are largely consistent with prior studies,^16,17,19–23^ with the exception of a report by Magann et al^18^ that reported a decreased rate of postpartum hemorrhage in women with resolved low placentation compared with women with a normally located placenta. Of note, our group has previously reported a similarly high rate of postpartum hemorrhage between women with a low-lying placenta resolved during the late third trimester (14%) and those with a persistent low placentation at the time of delivery (18.5%).^13^

Analysis of postpartum hemorrhage and related outcomes according to the timing of low placentation resolution noted progressively increasing risks, with people with later resolution experiencing higher odds of postpartum hemorrhage of 1,000 mL or more, second-line uterotonic drugs, and tranexamic acid compared with people with earlier low placentation resolution. These results are consistent with those reported by DeBolt and colleagues,^16^ who investigated postpartum hemorrhage and postpartum hemorrhage–related maternal morbidity in relation to the type of low placentation at the midtrimester scan. Of note, in our cohort, late resolution occurred more frequently among people with placenta previa diagnosed in the midtrimester than those with a low-lying placenta initially observed.

We also identified a higher rate of induction of labor, mostly for rupture of membranes at term for more than 24 hours in women with resolved low placentation compared with those with a normally located placenta, which is a novel and unexpected finding. A possible explanation might be that trophotropism, the mechanism underlying the process of low placentation resolution, can weaken the amniotic membrane structure in close proximity to the placental regions that undergo atrophy, thus leading to a higher incidence of rupture of membranes before labor onset. In their retrospective study, Kim and coauthors^21^ identified an increased frequency of induction of labor for bleeding among women with resolved low placentation; however, they did not report the overall rate of induction of labor or other indications for this intervention.

The main strength of our study was its prospective multicenter design. A standardized use of transvaginal ultrasonography at the midtrimester scan to confirm placental location by expert physicians and follow-up transvaginal ultrasonography assessments to evaluate resolution of low placentation ensured reliability of diagnosis. We used a universally recognized definition of low placentation of a placental edge overlapping the internal os or at 1–20 mm from it. We quantitatively assessed blood loss in all women and evaluated several additional secondary outcomes related to postpartum hemorrhage, thus strengthening the reliability of our results. However, our study did have limitations. It was conducted at academic maternity centers involved in a regional maternal–fetal medicine network and with a low rate of postpartum hemorrhage, possibly limiting the generalizability of our findings to other settings. In addition, although our study was prospectively conducted and we recruited an appropriate number of women to explore the primary outcome of interest, our study was not powered to investigate outcomes in subgroups defined by the timing of low placentation resolution. Thus, a larger sample size would be advisable to elucidate the odds of postpartum hemorrhage and related morbidity among women with early and late resolution of low placentation. Additional investigation would also be helpful in better understanding the observed association between low placentation resolution and rupture of membranes at term.

In conclusion, our study provides high-quality evidence to support the integration of resolved low placentation among factors known to increase the likelihood of postpartum hemorrhage, thus improving the identification of women at risk at the time of hospital admission for childbirth and optimizing care planning.
